# Supplementary material for: Terahertz response of monolayer and few-layer WTe2 at the nanoscale
Source: Nat Commun. 2021 Sep 22;12:5594. doi: 10.1038/s41467-021-23933-z (PMC8458490; doi:10.1038/s41467-021-23933-z)
Supplement: Supplementary file 1 — Supplementary Information [file 41467_2021_23933_MOESM1_ESM.pdf]

Supplementary Information for Ran, et al., "Terahertz response of monolayer and few layer WTe_2 at the nanoscale".

Supplementary Note 1: Comparison between topography and near-field images

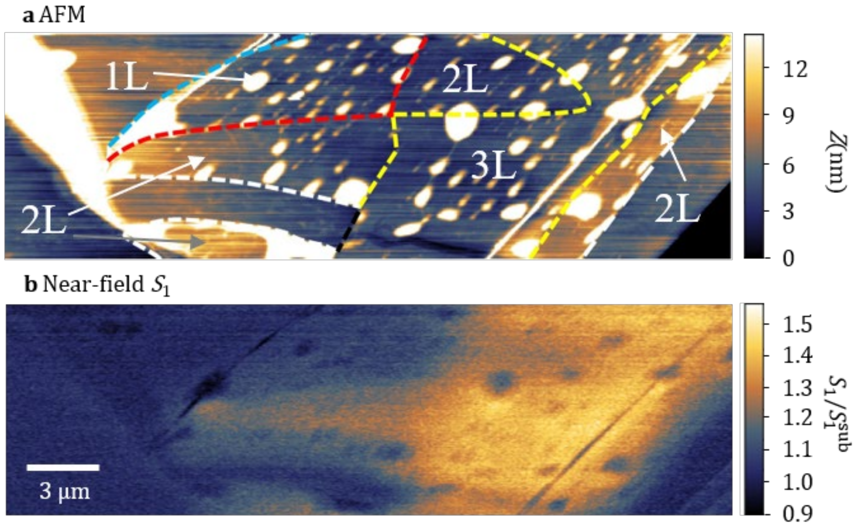

Supplementary Figure 1|Comparison between topography and near-field images. a The topography image of the field-of-view shown in Fig. 1b and c of the main text. The dashed lines, indicating the boundaries between different regions, are determined by topographical contrast of monolayer (1L), bilayer (2L) and tri-layer (3L) regions. **b** The near-field S_1 image taken simultaneously with the topography image.

A side-by-side comparison between topography and near-field images helps better locate different regions on the sample surface. The micrometer-sized white dots in topography images, corresponding to the black dots in the near-field images, result from bubbles between WTe_2 sample and hBN encapsulation layers. Because the existence of the top layer hBN and bubbles, locating different regions in atomic force microscopy (AFM) images is challenging. Nevertheless, we can still track the boundary by taking line-cuts and compare with the near-field image and the optical inspection image.

Supplementary Note 2. Spatial resolution and approach Curve of S_1 and S_2 near-field signal

To illustrate how localized the signal is above the sample surface, we measured the dependence of near-field signal on the tip-sample distance. In the Supplementary Figure 2, S_2 signal is much more localized than S_1. More than 90% of S_2 signal is contributed within 150nm above the sample surface. For S_1 signal, this length scale of signal decay is ~500nm. In terms of full width half maximum, the decay length scale for S_1 and S_2 signal are <100nm and <50nm. The in-plane length scale of E-field localization of near-field signal equals that of out-of-plan [1]. Hence, the resolution of S_1 (S_2) image is no larger than 200nm (100nm).

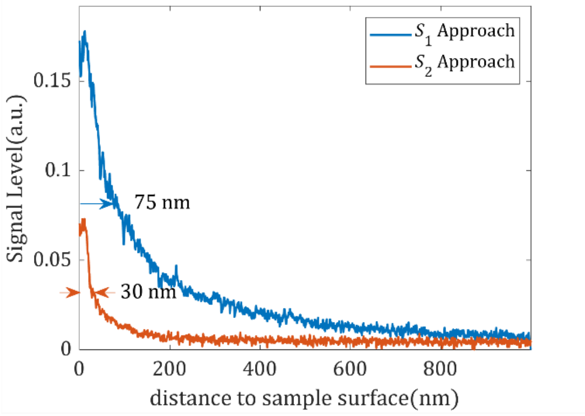

Supplementary Figure 2|Near-field Approach Curve of S_1 and S_2 signal. The approach curve is measured by varying the tip-sample distance. S_1 signal is mainly contributed by E field within 500nm above the sample surface, whereas S_2 signal is confined within 150nm above the sample surface.

Supplementary Note 3: Zoomed-in images of monolayer WTe_2

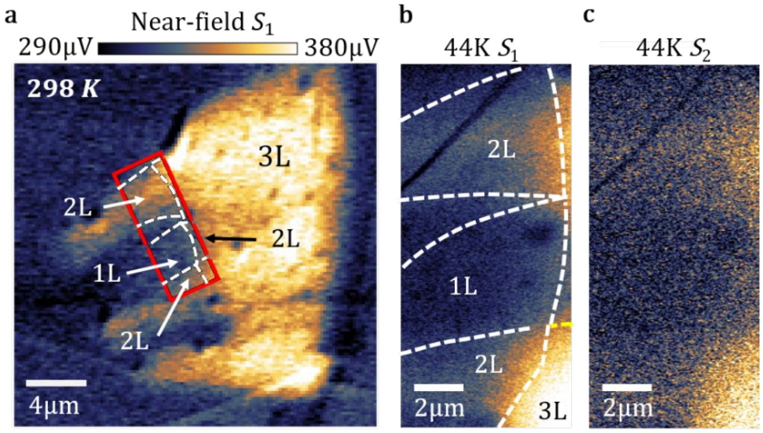

Supplementary Figure 3|Zoomed-in images of monolayer WTe_2. a THz near-field S_1 image of the whole sample area at room temperature. We mark the boundaries of terraces with different number of WTe_2 layers (1L, 2L, 3L) with dashed lines. The red frame indicates the area where the monolayer images are taken. **b** Zoomed-in S_1 image around monolayer region. **c** Zoomed-in S_2 image around monolayer region.

Monolayer WTe_2 is confirmed to support quantum spin Hall state below 100K [2] [3] [4] [5] [6] [7] with edge conduction channels. At the lowest temperature of the experiment 44K, THz response near images around monolayer WTe_2 was measured in more detail (Supplementary Figure 3). The location of the zoomed-in field-of-view is indicated in Supplementary Figure 3a with a red frame. In this field-of-view, we can see the boundaries between monolayer WTe_2 and substrate and between monolayer and bi-layer WTe_2. With our current signal to noise ratio, no clear feature arising from the topological edge state is observed, despite previous observation of the edge state at DC [8] and microwave frequencies [3]. It is possible that the increase of conductivity due to edge state does not extend to THz range. However, the low conductivity [8] of the edge state is also challenging for nano-THz technology.

Supplementary Note 4: The influence of in-plane anisotropy of WTe_2 on near-field modeling.

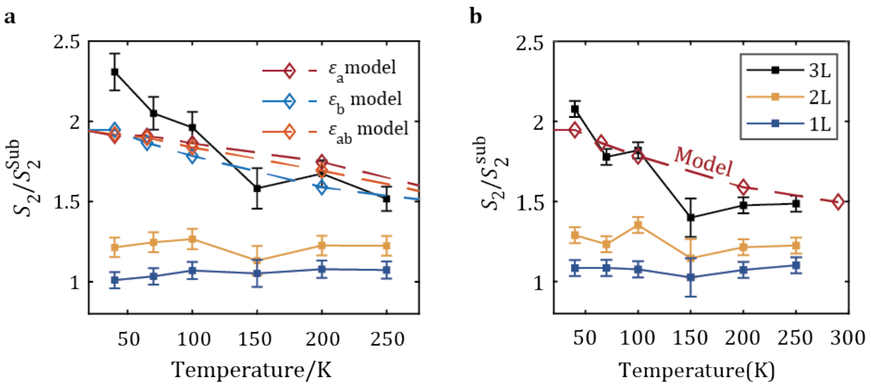

Supplementary Figure 4| a Temperature dependent normalized S_2 from experiment and modeling. Experimental data points are indicated with solid squares. Near-field modeling are indicated with empty diamonds. Three different sets of parameters: $\epsilon_{\parallel} = \epsilon_b$, $\epsilon_{\parallel} = \epsilon_a$ and $\epsilon_{\parallel} = (\epsilon_a + \epsilon_b)/2$ are configured for 3L WTe_2 modeling. **b** Normalized S_2 signals averaged in the whole regions of 1L, 2L and 3L. The boundaries of corresponding regions are indicated in Fig. 2a in the main text.

The near-field modeling mainly follows the procedure described in Ref. [1]. One assumption is that the material can be treated as isotropic or uniaxial material with a unique out-of-plane axis. On the contrary, WTe_2 reveals notable in-plane anisotropy with distinct plasma frequencies between a and b axes within the WTe_2 plane [9] [10]. We assumed that tri-layer WTe_2 can be reasonably described as a uniaxial material with its in-plane relative permittivity represented by that of b-axis of bulk WTe_2. Nevertheless, the finite in-plane anisotropy is evident only at much higher frequencies (~12 THz) [9] and gives rise to minor quantitative change in the observed near-field response, as demonstrated below. We first performed calculations with both a purely a-axis response and an effective dielectric function averaging between a-axis and b-axis data. In Supplementary Figure 4, three different configurations are displayed. Modeling with $\epsilon_{\parallel} = \epsilon_{\mathrm{b}}$ has the highest increase of near-field signal at low temperature. The increase is lower in $\epsilon_{\parallel} = (\epsilon_{\mathrm{b}} + \epsilon_a)/2$ and the lowest in $\epsilon_{\parallel} = \epsilon_{\mathrm{a}}$, despite the overall differences are small enough to neglect.

The temperature dependent behavior is not unique to the field of view we chose to perform averaging. In Supplementary Figure 4b, we display the near-field signal averaged within the entire regions of 1L, 2L and 3L indicated in Fig. 2a in the main text. Because the edges with lower signal are included, the overall signal level is decreased. The inclusion of bubbles and other local defects contributes to slightly worse statistics. Except for the decreased signal level and influence due to defects, the temperature dependent behavior of each region is not changed.

Supplementary Note 5: Mid-IR response of monolayer and few layer WTe2 at the nanoscale.

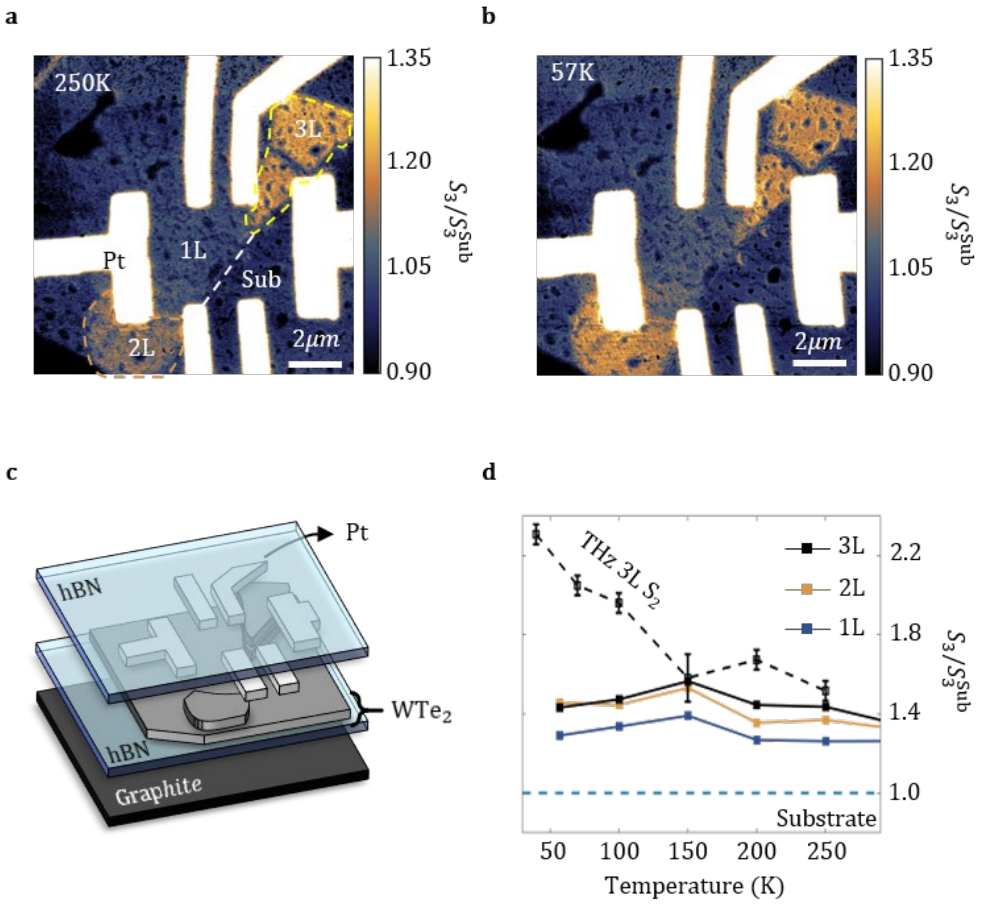

Supplementary Figure 5|Near-field nano-imaging on multi-terraced encapsulated WTe_2 micro-crystal in the mid-infrared. Image of the normalized near-field S_3 signal (ω=27 THz) at **a** 250 K and **b** 57K. **c** Layout of the sample measured in the mid-IR near-field experiments. **d** Temperature dependence of mid-IR near-field signal for regions with different number of layers.

For completeness, we also acquired near-field data in the frequency range higher than the plasma frequency of bulk WTe_2. The experiment is carried out in the mid-IR range with a 27 THz CO_2 laser. Here, a third (S_3) or higher harmonic of the near-field signal must be measured to suppress the far-field contribution [11]. The WTe_2-based structure investigated in this experiment shares common elements with the devices in the main text Fig.1a. The major difference is that there is another layer of graphite in between the SiO_2/Si substrate and bottom layer hBN. This latter architecture is only suitable for mid-IR experiments: the graphite beneath the sample saturates the near-field signal in THz range but not in mid-IR range. Images acquired at 250K and 57K are shown in Supplementary Figure 4a&b. The temperature dependence of nano-IR signals is plotted in Supplementary Figure 5d. WTe_2 shows a much weaker nano-IR signal compared to the signal produced by the platinum electrodes. Regions with more WTe_2 layers show systematically higher mid-IR signal levels and the temperature dependence for all layers is insignificant

(Supplementary Figure 5d). This temperature independent behavior is well explained by our model result shown in the main text Fig. 3d.

Supplementary Note 6: Near-field electrodynamics of thermally activated carriers of bi-layer and thicker WTe_2

In calculating the near-field signal of bi-layer WTe_2, we mainly consider the Drude response of its thermally activated carriers. The carrier density is computed assuming WTe_2 has quadratic bands with effective electron mass at the conduction and valence band edge:

$$\mathrm{n_{2D}} = \int_{\mathrm{E_C-E_F}}^{\infty} 2\mathrm{f_{FD}(E)g_{2D}(E)dE} = \frac{2\mathrm{kTm_C^*}}{\pi\hbar^2}\ln\left(e^{-\frac{\mathrm{E_C-E_F}}{\mathrm{kT}}}+1\right)$$

$$\mathrm{p_{2D}} = \int_{|\mathrm{E_D-E_F}|}^{\infty} 2\mathrm{f_{FD}(E)g_{2D}(E)dE} = \frac{2\mathrm{kTm_V^*}}{\pi\hbar^2}\ln\left(e^{-\frac{|\mathrm{E_V-E_F}|}{\mathrm{kT}}}+1\right)$$

Here, m_C^* and m_V^* are the effective electron mass at the band edge of conduction and valence band. E_C, E_V and E_F are the energy of conduction band edge, valence band edge and Fermi energy of bi-layer WTe_2. The Fermi energy is determined by the neutrality condition $\mathrm{n_{2D}} = \mathrm{p_{2D}}$ of the investigated system. The permittivity of the model bi-layer:

$$\epsilon = \epsilon_\infty - \frac{\omega_P^2}{\omega(\omega - i\gamma)}$$

Here ω_P is the plasma frequency determined by the thermally activated carrier density:

$$\omega_{p,n} = \sqrt{\frac{n_{2d}e^2}{dm^*\epsilon'}} = \sqrt{\frac{2e^2kT}{d\pi\hbar^2\epsilon'\epsilon_0}ln\left(e^{-\frac{E_C-E_F}{kT}}+1\right)}$$

$$\omega_{p,p} = \sqrt{\frac{p_{2d}e^2}{dm^*\epsilon'}} = \sqrt{\frac{2e^2kT}{d\pi\hbar^2\epsilon'\epsilon_0}ln\left(e^{-\frac{|\mathrm{E_V-E_F}|}{\mathrm{kT}}}+1\right)}$$

Here $\mathrm{d} = 1.4$ nm, $\epsilon' = 2.2$ is the dielectric constant of hBN at THz frequencies. The scattering rate γ of the model bi-layer is assumed to be the average of the two scattering rates of two Drude components in the bulk [12] $\gamma = (\gamma_1 + \gamma_2)/2$.

For thicker samples, the carrier density multiplies due to the increase of the number of the electronic bands. In the calculation of ω_p, the increase of carrier density cancels the increase of thickness. Therefore, the ω_p maintained the same value for WTe_2 of different thicknesses.

Supplementary Note 7: Temperature dependence of white-light near-field signal produced by LRM.

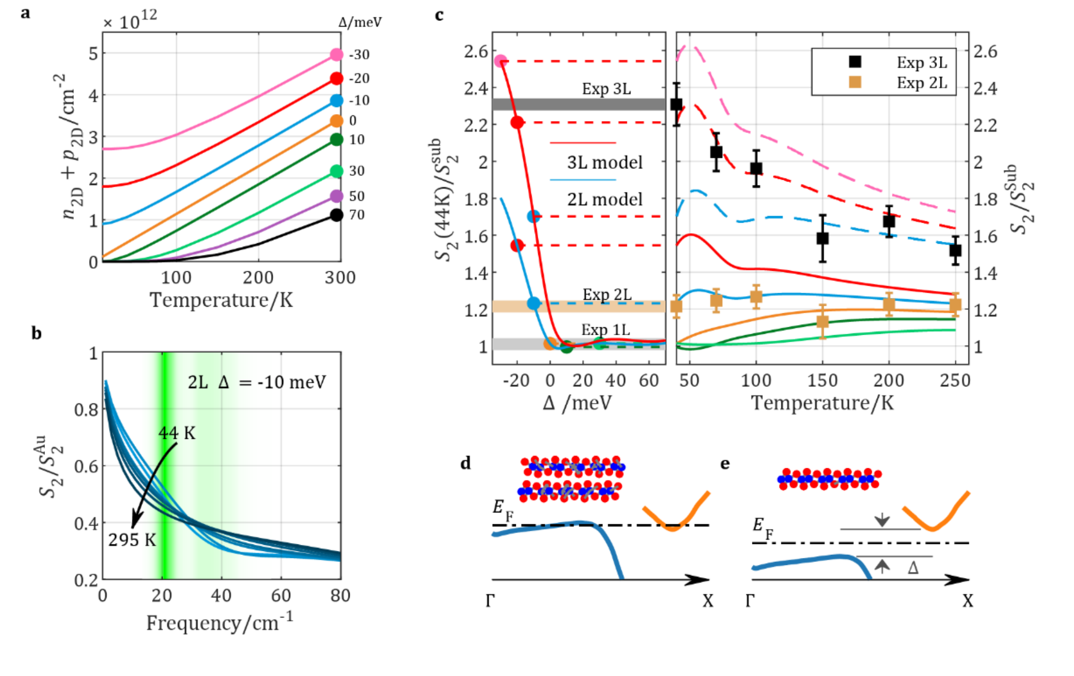

Supplementary Figure 6|Near-field electrodynamics of thermally activated carrier of few-layer WTe_2. a Temperature dependence of thermally activated carrier densities at different gap sizes calculated (Supplementary Note 5) based on the band structure investigated by ARPES [13]. **b** Near-field spectroscopic response of the thermally activated carriers of a model 2L WTe2 with $\Delta = -10\ meV$. The green shaded region represents the power spectrum of the THz probe. **c** Right panel: Temperature dependent WL signal calculation based on LRM for 2L (solid lines) and 3L (dashed lines) WTe_2 with gap sizes ranging from -30 meV to +30 meV. Along with the model, nano-THz data of 1L, 2L and 3L WTe_2 are displayed with squares. Both the model curves and experiment points are normalized to the substrate value. Left panel: The gap-size dependent near-field signal of 2L (blue) and 3L (red) at 44 K. The colored dots on the curves corresponds to the curves shown in the right panel. **d, e** Hypothetical band structure of semimetallic 2L WTe_2 (left) and insulating 1L WTe_2 *[13]*with a bandgap Δ>60 meV (right).

In Supplementary Figure 6c, we show temperature dependence of near-field signal on 2L and 3L WTe_2 with different gap-size produced by lightning-Rod model. For 2L (solid line in Supplementary Figure 6c), if the gap size is positive and large, the signal at all temperature is close to the substrate. When the gap size is close to 0, the signal is still strongly suppressed at low temperature due to the low carrier density. When the gap becomes negative, the low temperature signal quickly increases and is even higher than high temperature when the gap size is below -10 meV. When the thickness increases from 2L to 3L (dashed line in Supplementary Figure 6c), the overall signal level is increased. With the increase of the gap size in the negative direction, the signal becomes stronger. We found Δ~-10 meV and Δ~-20 meV almost perfectly matches the 2L and 3L data, correspondingly.

Supplementary Note 8: Real-space near-field modeling of SPP structures

To generate predicted real-space images shown in the main text, we apply a semi-analytic method that approximates the near-field scattering signal from a 2D material as proportionate (to first-order) by the z-polarization of a polarizable dipole raster-scanned (at a height $z = z_{\mathrm{dp}}$) some tens of nanometers over the surface of a sample (at $z = 0$):

$$S(\boldsymbol{\rho}_{\mathrm{dp}}) \sim p_z \approx \alpha E_{\mathrm{ref},z}\left(\boldsymbol{\rho}_{\mathrm{dp}}, z = z_{\mathrm{dp}}\right). \qquad \text{(S1)}$$

Here α denotes the dipole polarizability, $E_{\mathrm{ref},z}$ denotes the z-component of the electric near-field reflected by the sample in response to the incident dipole field, and $\boldsymbol{\rho}_{\mathrm{dp}}$ denotes evaluation at the in-plane coordinate of the probe. Although this expression represents only the first term in a Born expansion of the full self-consistent dipole polarization [14] a similar conceptual treatment was previously shown to faithfully replicate the polaritonic near-field response of two-dimensional materials as measured by scanning near-field optical microscopy [15]. Here we summarize the key points enabling our calculation of Eq. S1 in the quasi-electrostatic approximation and defer more detailed discussion to forthcoming work.

We recast Eq. S1 in a form reminiscent of the local photonic density of states [16] measured at the location $\boldsymbol{r}_{\mathrm{dp}} = (\boldsymbol{\rho}_{\mathrm{dp}}, z_{\mathrm{dp}})$ of our dipole probe:

$$S(\boldsymbol{\rho}_{\mathrm{dp}}) \sim \int_{z>0} dV\ \hat{\jmath}_{\mathrm{dp}} \cdot \vec{E}_{\mathrm{ref}} = \int_{z>0} dV\ \nabla \cdot \hat{\jmath}_{\mathrm{dp}}\, \Phi_{\mathrm{ref}} \propto \int_{z>0} dV\ -\varrho_{\mathrm{dp}} \cdot \Phi_{\mathrm{ref}} \qquad \text{(S2)}$$

Here $\hat{\jmath}_{\mathrm{dp}}$ denotes the unit vector oriented along the direction of the point dipole current, ϱ_{dp} denotes the instantaneous charge distribution associated with the dipole, and Φ_{ref} is the electrostatic potential for the reflected field given by $\vec{E}_{\mathrm{ref}} = -\nabla\Phi_{\mathrm{ref}}$. Now $S(\boldsymbol{\rho}_{\mathrm{dp}})$ can be evaluated entirely in the plane $z = 0$ by identifying the "incident" electrostatic potential generated by the dipole through $\varrho_{\mathrm{dp}} = -\frac{1}{4\pi}\nabla^2\Phi_{\mathrm{dp}}$ and integrating Eq. S2 by parts, yielding:

$$\int_{z>0} dV\ -\varrho_{\mathrm{dp}} \cdot \Phi_{\mathrm{ref}} = \frac{1}{4\pi}\left[\int_{z=0^+} dA\ \left(-\hat{z} \cdot \nabla\Phi_{\mathrm{dp}}\right)\Phi_{\mathrm{ref}} - \int_{z>0} dV\, \nabla\Phi_{\mathrm{dp}} \cdot \nabla\Phi_{\mathrm{ref}}\right]$$
$$= \frac{1}{4\pi}\int_{z=0^+} dA\ \left(\Phi_{\mathrm{dp}}\partial_z\Phi_{\mathrm{ref}} - \partial_z\Phi_{\mathrm{dp}}\Phi_{\mathrm{ref}}\right). \qquad \text{(S3)}$$

Here we have applied the source-free condition $\nabla^2\Phi_{\mathrm{ref}} = 0$ in the volume $z > 0$. Eq. (S3) represents an approximation for the signal $S(\boldsymbol{\rho}_{\mathrm{dp}})$ when Φ_{dp} is produced from a dipole-like probe at $\boldsymbol{r}_{\mathrm{dp}}$. Further simplification is admitted by the fact that $\Phi_{\mathrm{ref}} = -\hat{R}\Phi_{\mathrm{dp}} \equiv -\Phi_{\mathrm{R}}$, with $\hat{R}$ a generalized reflection operator. Moreover, for scalar potentials $\Phi_{1,2}$ harmonic (*viz.* source-free) in the plane of integration, $\int dA\, \Phi_1\partial_z\Phi_2 = \pm\int d^2q\ |\boldsymbol{q}|\ \widetilde{\Phi}_1\widetilde{\Phi}_2$, where tilde quantities represent in-plane Fourier transforms with respect to the momentum $\boldsymbol{q}$, and $\pm$ correspond to the cases where Φ_2 is sourced from $z > 0$ or $z < 0$, respectively. With these considerations, Eq. (S3) reduces to:

$$S(\boldsymbol{\rho}_{\mathrm{dp}}) \sim \frac{1}{2\pi}\int d^2q\,|\boldsymbol{q}|\,\widetilde{\Phi}_{\mathrm{dp}}\widetilde{\Phi}_R = \frac{1}{2\pi}\int dA\,(q * \Phi_{\mathrm{dp}})\hat{R}\Phi_{\mathrm{dp}} \qquad \text{(S4)}$$

where $(q * \Phi_{\mathrm{dp}})$ represents the incident scalar potential spatially convolved at $z = 0^+$ with a sharpening function with Fourier kernel $|\boldsymbol{q}|$. Eq. (S4) represents a norm of the function Φ_{dp} in the plane $z = 0$ with respect to the composite reflection operator $q * \hat{R}$. By way of demonstration, we can consider cases where the reflected field is given by $\widetilde{\Phi}_R = r_p(q)\widetilde{\Phi}_{\mathrm{dp}}(q)$, with r_p the momentum-resolved Fresnel coefficient for *e.g.* a layered medium with in-plane translational invariance. Applying the in-plane Fourier transform of the dipole potential $\widetilde{\Phi}_{\mathrm{dp}}(\boldsymbol{q}) = \mathrm{e}^{-qz_{dp}}$ at $z = 0$, for such cases Eq. S4 evaluates to $S \propto \int dq\, r_p(q)\, q^2 e^{-2qz_{dp}}$. This is indeed the first-order term in a Born series expansion of the point dipole model widely used to predict near-field observables in the case of multilayered systems [17] [18]. Meanwhile, whereas the real-space counterpart that we present in Eq. S4 remains underreported, it provides a powerful means to predict images recorded by scanning near-field optical microscopy.

We now briefly describe our method for evaluating $\hat{R}\Phi_{\mathrm{dp}}$ in the case of a spatially inhomogeneous 2D material at $z = 0$ described by a (piecewise) optical conductivity $\sigma_{2D}(\boldsymbol{\rho})$ upon a substrate with isotropic reflectivity β_{subs}. We first consider the integro-differential equation for the scalar potential Φ_{ref} generated by σ_{2D} in response to the potential Φ_{dp} of our quasi-dipolar probe [19], in absence of a substrate:

$$\left[1 + V * \sum_m \frac{1}{2\pi q_{p,m}}\nabla\cdot\bar{\sigma}_m(\boldsymbol{\rho})\,\nabla\right]\Phi(\boldsymbol{\rho}) = \Phi_{\mathrm{dp}}(\boldsymbol{\rho}),\ \text{with}\ \Phi = \Phi_{\mathrm{dp}} + \Phi_{\mathrm{ref}}. \qquad \text{(S5)}$$

Here m indexes the piecewise homogeneous domains of our 2D material (*i.e.* in our case domains of mono-, bi-, and tri-layer WTe_2), $q_{p,m}$ denotes the complex plasmon wavevector associated with each domain, and $\bar{\sigma}_m(\boldsymbol{\rho})$ are piecewise homogeneous functions equal to zero or 1 marking the lateral regions $\boldsymbol{\rho} \in \Omega_m$ occupied by each domain. Meanwhile, $V(\boldsymbol{r},\boldsymbol{r}') = 1/|\boldsymbol{r} - \boldsymbol{r}'|$ is the Coulomb kernel, and the asterisk $(*)$ denotes spatial convolution over the in-plane coordinate $\boldsymbol{\rho} = (x, y)$. We solve Eq. S5 by expanding $\Phi_{\mathrm{ref}}(\boldsymbol{\rho}) = \sum_{mn}\phi_{mn}^{\mathrm{ref}}\Phi_{mn}(\boldsymbol{\rho})$ into an orthonormal basis of eigenfunctions specified on the domains $\partial\Omega_m$ by $\nabla\cdot\bar{\sigma}_m(\boldsymbol{\rho})\,\nabla\Phi_{mn}(\boldsymbol{\rho}) = -q_{mn}^2\Phi_{mn}(\boldsymbol{\rho})$ and subject to the "zero current" boundary conditions $\hat{n}\cdot\nabla\Phi_{mn}$ on the domain edges $\partial\Omega_m$. These functions are obtained with the finite element solver FEniCs [20] after meshing the experimentally relevant domain configurations shown in Figure 5a of the main text.

Values for the plasmon wave-vectors are inferred from the respective layer thicknesses d_m and complex in-plane optical permittivities ε_m of each domain in Ω_m according to $q_{p,m} = 2(1-\varepsilon_m)^{-1}\, d_m^{-1}$; see Supplementary Table 1 for the values used in our simulations. Although WTe_2 is known to exhibit biaxial in-plane permittivity, for simplicity in our simulations we apply an isotropic approximation as earlier discussed in this supplement.

Domain (indexed)	Thickness (nm)	Optical permittivity (THz)	Plasmon wave-vector (cm^{-1})
Monolayer (70 K)	0.7	41	-710000
Monolayer (295 K)	0.7	41	-710000
Bilayer (70 K)	1.4	-463+ 1389i	3091+9252i
Bilayer (295 K)	1.4	-110 + 1362i	849+10420i
Trilayer (70 K)	2.1	-677 + 2031i	1409+4219i
Trilayer (295 K)	2.1	-134 + 1576i	514+6000i

Supplementary Table 1| Parameters for modeling real-space near-field images of WTe_2 monolayer, bilayer, and trilayer domains at 70 K and 295 K. Layer thickness and the complex-valued optical permittivity $\varepsilon=\varepsilon_1+i\varepsilon_2$ determines the plasmon wave-vector. Permittivities are estimated according to discussion in the main test.

Assembling the expansion coefficients ϕ_{mn}^{ref} into a vector $\boldsymbol{\phi}_{\text{ref}}$ (taking mn as a composite index), we solve Eq. (S5) by the matrix equation:

$$\boldsymbol{\phi}_{\text{ref}} = -\left[\frac{-\boldsymbol{V}\sum_m \boldsymbol{q}_m^2/(2\pi q_{p,m})}{1-\boldsymbol{V}\sum_m \boldsymbol{q}_m^2/(2\pi q_{p,m})}\right]\boldsymbol{\phi}_{dp} \tag{S6}$$

As with $\boldsymbol{\phi}_{\text{ref}}$, here $\boldsymbol{\phi}_{dp}$ represents the vector of expansion coefficients for $\Phi_{\text{dp}}(\boldsymbol{\rho})$. Meanwhile, each $\boldsymbol{q}_m^2$ denotes a diagonal matrix of eigenvalues q_{mn}^2 acting in the vector subspace spanned by $\{\Phi_{mn}\ \forall n\}$ at fixed domain index m, and $\boldsymbol{V}$ is the coulomb matrix whose elements are given by $V_{kl,mn} = \int_{z=0} dA\ \Phi_{kl}(\boldsymbol{\rho})\ V * \Phi_{mn}(\boldsymbol{\rho})$. The term in brackets in Eq. (S6) represents the generalized reflection operator $\boldsymbol{R}$ for the system in the Φ_{mn} basis. (The denominator is understood in the sense of a matrix inverse applied before pre-multiplication by the numerator.) While we defer the derivation to forthcoming work, this reflection operator generalizes to the case of our 2D materials upon a substrate with isotropic reflectivity β_{subs} as follows:

$$\boldsymbol{R} = \frac{\beta_{\text{subs}} - \boldsymbol{V}\sum_n \boldsymbol{q}_n^2/(2\pi\kappa q_{p,n})}{1-\boldsymbol{V}\sum_n \boldsymbol{q}_n^2/(2\pi\kappa q_{p,n})} \tag{S7}$$

Here $\kappa = (\varepsilon_{\text{subs}}+1)/2$ and $\beta_{\text{subs}} = (\varepsilon_{\text{subs}}-1)/(\varepsilon_{\text{subs}}+1)$, with $\varepsilon_{\text{subs}}$ the substrate permittivity; in this work the substrate includes 20nm hBN and bulk SiO_2.

We also define a symmetric matrix $\boldsymbol{Q}$ in the Φ_{mn} basis corresponding to the spatial convolution in Eq. S4, with elements given by $Q_{kl,mn} = \int_{z=0} dA\ \Phi_{kl}(\boldsymbol{\rho})\ q * \Phi_{mn}(\boldsymbol{\rho})$. Since the Φ_{mn} are orthonormal, Eq. S4 reduces to:

$$S(\boldsymbol{\rho}_{\rm dp}) \sim \frac{1}{2\pi} \boldsymbol{\phi}_{dp}(\boldsymbol{\rho}_{\rm dp})^T \, \boldsymbol{QR} \, \boldsymbol{\phi}_{dp}(\boldsymbol{\rho}_{\rm dp}). \qquad \text{(S8)}$$

This represents a vector norm of $\boldsymbol{\phi}_{dp}$ with respect to the matrix $\boldsymbol{QR}$.

In summary, after computing eigenfunctions Φ_{mn} associated with our sample geometry, we compute symmetric matrices $\boldsymbol{V}$ and $\boldsymbol{Q}$ and the generalized reflectance operator $\boldsymbol{R}$. Then, in order to predict a spatial map $S(\boldsymbol{\rho}_{\rm dp})$, we simply project the incident potential emitted by our quasi-dipolar probe at each location $\boldsymbol{\rho}_{\rm dp}$ into the Φ_{nm} basis by evaluating the vector of coefficients $\phi_{dp,mn}(\boldsymbol{\rho}_{\rm dp}) = \int dA \; \Phi_{\rm dp}(\boldsymbol{\rho})\Phi_{mn}(\boldsymbol{\rho})$ and successively applying Eq. S8. Although the eigenbasis $\{\Phi_{mn}\}$ is of infinite size, projections into $\boldsymbol{\phi}_{dp}$ decay exponentially with n when Φ_{mn} are sorted by increasing eigenvalue q_{mn}^2, so a truncated basis of size $N \approx 10^3$ is in our case sufficient for a converged map of near-field scattering amplitude $|S(\boldsymbol{\rho}_{\rm dp})|$. In this way, the observables of near-field microscopy can be predicted entirely by evaluating functions in the plane of the sample ($z = 0$). This computational method may be suitable for qualitative and quantitative modeling of near-field response of other spatially inhomogeneous 2D heterostructures. Such applications and details of their unique numerical implementation will be reported elsewhere.

References

[1] A. S. McLeod, P. Kelly, M. D. Goldflam, Z. Gainsforth, A. J. Westphal, G. Dominguez, M. H. Thiemens, M. M. Fogler and D. N. Basov, "Model for quantitative tip-enhanced spectroscopy and the extraction of nanoscale-resolved optical constants," *Physical Review B,* vol. 90, p. 085136, 2014.

[2] Z. Fei, M. D. Goldflam, J.-S. Wu, S. Dai, M. Wagner, A. S. McLeod, M. K. Liu, K. W. Post, S. Zhu, G. C. A. M. Janssen and others, "Edge and surface plasmons in graphene nanoribbons," *Nano letters,* vol. 15, p. 8271–8276, 2015.

[3] Y. Shi, J. Kahn, B. Niu, Z. Fei, B. Sun, X. Cai, B. A. Francisco, D. Wu, Z.-X. Shen, X. Xu and others, "Imaging quantum spin Hall edges in monolayer WTe2," *Science advances,* vol. 5, p. eaat8799, 2019.

[4] L. Peng, Y. Yuan, G. Li, X. Yang, J.-J. Xian, C.-J. Yi, Y.-G. Shi and Y.-S. Fu, "Observation of topological states residing at step edges of WTe 2," *Nature communications,* vol. 8, p. 659, 2017.

[5] S. Tang, C. Zhang, D. Wong, Z. Pedramrazi, H.-Z. Tsai, C. Jia, B. Moritz, M. Claassen, H. Ryu, S. Kahn and others, "Quantum spin Hall state in monolayer 1T'-WTe 2," *Nature Physics,* vol. 13, p. 683, 2017.

[6] S. Wu, V. Fatemi, Q. D. Gibson, K. Watanabe, T. Taniguchi, R. J. Cava and P. Jarillo-Herrero, "Observation of the quantum spin Hall effect up to 100 kelvin in a monolayer crystal," *Science,* vol. 359, p. 76–79, 2018.

[7] X. Qian, J. Liu, L. Fu and J. Li, "Quantum spin Hall effect in two-dimensional transition metal dichalcogenides," *Science,* vol. 346, p. 1344–1347, 2014.

[8] Z. Fei, T. Palomaki, S. Wu, W. Zhao, X. Cai, B. Sun, P. Nguyen, J. Finney, X. Xu and D. H. Cobden, "Edge conduction in monolayer WTe 2," *Nature Physics,* vol. 13, p. 677, 2017.

[9] A. J. Frenzel, C. C. Homes, Q. D. Gibson, Y. M. Shao, K. W. Post, A. Charnukha, R. J. Cava and D. N. Basov, "Anisotropic electrodynamics of type-II Weyl semimetal candidate WTe 2," *Physical Review B,* vol. 95, p. 245140, 2017.

[10] C. Wang, S. Huang, Q. Xing, Y. Xie, C. Song, F. Wang and H. Yan, "Van der Waals thin films of WTe 2 for natural hyperbolic plasmonic surfaces," *Nature communications,* vol. 11, p. 1–9, 2020.

[11] A. J. Sternbach, J. Hinton, T. Slusar, A. S. McLeod, M. K. Liu, A. Frenzel, M. Wagner, R. Iraheta, F. Keilmann, A. Leitenstorfer and others, "Artifact free time resolved near-field spectroscopy," *Optics Express,* vol. 25, p. 28589–28611, 2017.

[12] C. C. Homes, M. N. Ali and R. J. Cava, "Optical properties of the perfectly compensated semimetal WTe 2," *Physical Review B,* vol. 92, p. 161109, 2015.

[13] I. Cucchi, I. Gutiérrez-Lezama, E. Cappelli, S. McKeown Walker, F. Y. Bruno, G. Tenasini, L. Wang, N. Ubrig, C. Barreteau, E. Giannini and others, "Microfocus laser–angle-resolved photoemission on encapsulated mono-, bi-, and few-layer 1T′-WTe2," *Nano letters,* vol. 19, p. 554–560, 2018.

[14] R. Hillenbrand, B. Knoll and F. Keilmann, "Pure optical contrast in scattering-type scanning near-field microscopy," *Journal of microscopy,* vol. 202, p. 77–83, 2001.

[15] A. Y. Nikitin, P. Alonso-González, S. Vélez, S. Mastel, A. Centeno, A. Pesquera, A. Zurutuza, F. Casanova, L. E. Hueso, F. H. L. Koppens and others, "Real-space mapping of tailored sheet and edge plasmons in graphene nanoresonators," *Nature Photonics,* vol. 10, p. 239–243, 2016.

[16] L. Novotny and B. Hecht, Principles of nano-optics, Cambridge university press, 2012.

[17] Z. Fei, G. O. Andreev, W. Bao, L. M. Zhang, A. S. McLeod, C. Wang, M. K. Stewart, Z. Zhao, G. Dominguez, M. Thiemens and others, "Infrared nanoscopy of Dirac plasmons at the graphene–SiO2 interface," *Nano letters,* vol. 11, p. 4701–4705, 2011.

[18] J. Aizpurua, T. Taubner, F. J. G. de Abajo, M. Brehm and R. Hillenbrand, "Substrate-enhanced infrared near-field spectroscopy," *Optics Express,* vol. 16, p. 1529–1545, 2008.

[19] B. Rejaei and A. Khavasi, "Scattering of surface plasmons on graphene by a discontinuity in surface conductivity," *Journal of Optics,* vol. 17, p. 075002, 2015.

[20] M. Alnæs, J. Blechta, J. Hake, A. Johansson, B. Kehlet, A. Logg, C. Richardson, J. Ring, M. E. Rognes and G. N. Wells, "The FEniCS project version 1.5," *Archive of Numerical Software,* vol. 3, 2015.
